# The obligatory role of Activin A in the formation of heterotopic bone in Fibrodysplasia Ossificans Progressiva

**DOI:** 10.1016/j.bone.2017.06.011

**Published:** 2017-06-16

**Authors:** Dana M. Alessi Wolken, Vincent Idone, Sarah J. Hatsell, Paul B. Yu, Aris N. Economides

**Affiliations:** a Regeneron Pharmaceuticals, 777 Old Saw Mill River Road, Tarrytown, NY 10591, USA; b Division of Cardiovascular Medicine, Department of Medicine, Brigham and Women’s Hospital, Harvard Medical School, 75 Francis Street, Boston, MA 02115, USA; c Regeneron Genetics Center, 777 Old Saw Mill River Road, Tarrytown, NY 10591, USA

**Keywords:** Fibrodysplasia Ossificans Progressiva, Heterotopic Ossification, Activin A, ACVR1, Anti-Activin antibody, Progenitor cells

## Abstract

Fibrodysplasia Ossificans Progressiva (FOP) is a rare genetic disorder that presents at birth with only minor patterning defects, but manifests its debilitating pathology early in life with episodic, yet progressive and cumulative, heterotopic ossification (HO) of ligaments, tendons, and a subset of major skeletal muscles. The resulting HO lesions are endochondral in nature, and appear to be linked to inflammatory stimuli arising in association with known injuries, or from inflammation linked to normal tissue repair. FOP is caused by gain-of-function mutations in *ACVR1*, which encodes a type I BMP receptor. Initial studies on the pathogenic mechanism of FOP-causing mutations in *ACVR1* focused on the enhanced function of this receptor in response to certain BMP ligands, or independently of ligands, but did not directly address the fact that HO in FOP is episodic and inflammation-driven. Recently, we and others demonstrated that Activin A is an obligate factor for the initiation of HO in FOP, signaling aberrantly via mutant ACVR1 to transduce osteogenic signals and trigger heterotopic bone formation (Hatsell et al., 2015; Hino et al., 2015). Subsequently, we identified distinct tissue-resident mesenchymal progenitor cells residing in muscles and tendons that recognize Activin A as a pro-osteogenic signal (solely in the context of FOP-causing mutant *ACVR1*), and give rise to the cartilaginous anlagen that form heterotopic bone (Dey et al., 2016). During the course of these studies, we also found that the activity of FOP-causing *ACVR1* mutations does not by itself explain the triggered or inflammatory nature of HO in FOP, suggesting the importance of other, inflammation-introduced, factors or processes. This review presents a synthesis of these findings with a focus on the role of Activin A and inflammation in HO, and lays out perspectives for future research.

## 1. Introduction

Fibrodysplasia Ossificans Progressiva (FOP) (OMIM #135100) is a rare, mostly sporadic, autosomal-dominant disorder that is characterized by congenital skeletal dysplasias that are evident at birth or shortly thereafter [4–8], most notably malformation of the great toes [9]. However, in addition to these non-debilitating phenotypes, patients with FOP experience episodic heterotopic ossification (HO) of their tendons, ligaments, fascia, as well as a subset of skeletal muscles, and it is this aspect of FOP that is of utmost medical importance as it results in progressive and largely irreversible loss of mobility [7,10,11]. About half of the episodes of ossification are preceded by characteristic soft tissue swellings usually accompanied by pain and warmth, also referred to as “flareups” [11]. These episodes frequently occur following trauma, febrile illness, and other pro-inflammatory insults, suggesting that inflammation is a common trigger for HO [6,7,10,12]. The importance of inflammation in triggering HO in FOP is further supported by (**a**) reports that whenever these soft tissue swellings have been misdiagnosed as tumors and then either resected or biopsied, these procedures mostly resulted in more flare-ups and re-initiation of HO [6,10,13]; (**b**) histological evidence of immune cell infiltrates in ossifying lesions (with the caveat that it is not possible to decipher if this is a cause or an effect for any specific lesion due to lack of longitudinal data) [14–16]; (**c**) the apparent absence of HO events in a single FOP patient that was also the recipient of bone marrow transplantation (from his non-FOP sister) during the period of time that he was kept on immunosuppressive regimen, but recurrence of HO after he was taken off the immunosuppressive regimen [17]; and (**d**) observations using genetically accurate mouse models, in which “spontaneous” HO has been observed but wherein trauma, tissue damage, or other pro-inflammatory stimuli rapidly and reproducibly induce HO [1,3,18].

The first major advance in understanding the molecular mechanisms that underlie the pathophysiology of FOP was the identification of the causative gene – Activin A Receptor Type 1 (*ACVR1*) [19]. All FOP cases that have been sequenced to date arise from missense mutations in the cytoplasmic domain of ACVR1, indicating that there must exist a common molecular mechanism that is shared between them [20], even though there is some phenotypic variation in “non-classic” FOP cases [7,21–23]. Notably, the great majority of FOP cases share the same missense mutation – c.617G>A – which results in altering Arginine at position 206 to a Histidine (R206H). The discovery of the causative gene, together with the fact that more than 95% of FOP cases arise from one variant – *ACVR1[R206H]* – set in motion a lot of research aimed at deciphering the molecular mechanisms by which this mutant receptor drives HO. These studies postulated that ACVR1[R206H] brings about HO because it acts as a hyperactive receptor that exhibits both constitutive (i.e. ligand-independent) activity [19,24–26], as well as an enhanced response to ligands [25,27,28]. However, these conclusions were not derived from studies conducted in bona fide animal models of FOP, i.e. in the types of models that accurately capture both the genetics and the pathophysiology of FOP.

Given the apparent centrality of inflammation in the pathophysiology of FOP, we hypothesized that the process by which HO develops in this disease is likely to be ligand-dependent and to involve ligands that not only activate ACVR1[R206H] but that are also regulated by inflammation. In this way the dependence of HO on inflammation would be accounted for. In addition, we hypothesized that the inflammation-introduced signal(s) must be received by cells that can give rise to heterotopic bone via an endochondral process.

## 2. Review of role of Activin A in FOP

### 2.1. A genetically accurate mouse model of FOP

**Fig. 1.**
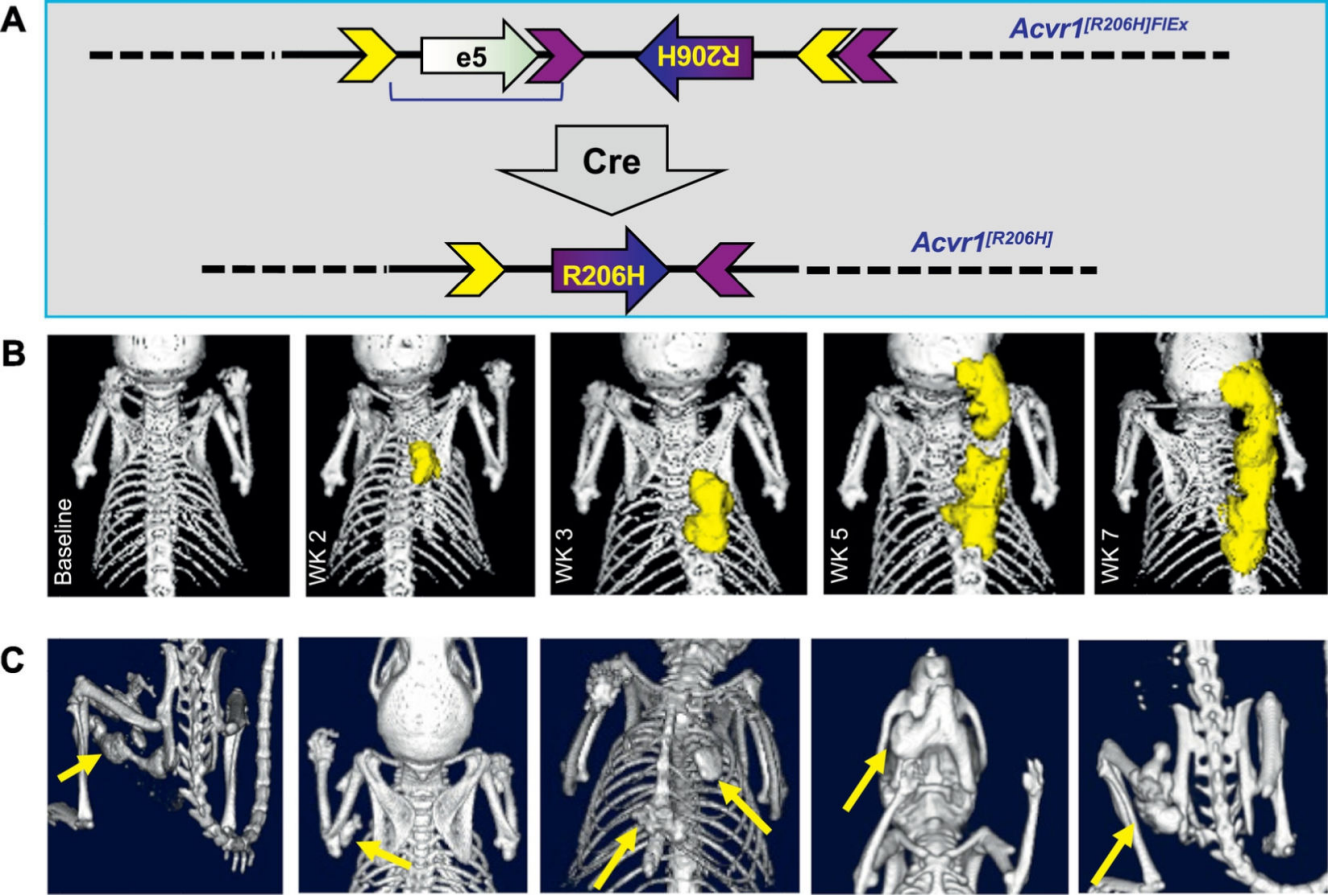
A genetically accurate and physiologically relevant mouse model of FOP. (A) *Acvr1*^*[R206H]FlEx*^ (*Acvr1*^*tm2.1Vlcg*^; MGI:5763014) [1] is a conditional-on knock-in allele of ACVR1[R206H]. It was generated by introducing the R206H variant in exon 5 of mouse *Acvr1*, and then placing this mutant exon in the antisense orientation within intron 5 of *Acvr1*. In order to restore the function of Acvr1, a wild type exon 5 from human *ACVR1* was placed upstream of the mutant exon (but in the sense strand), thereby preserving the structure of the resulting *Acvr1* transcript. These elements – wild type exon 5 and mutant exon 5 – were flanked by FlEx arrays [29] in a manner such that upon action of Cre, the wild type exon is deleted and mutant exon 5 is placed into the sense strand. Thereby, Cre effectively converts the *Acvr1*^*[R206H]FlEx*^ allele to *Acvr1*^*[R206H]*^, and hence recreates – in mice – the genotype found in *ACVR1*^*R206H*^ FOP patients. (B) HO (pseudocolored yellow) develops as early as 2 weeks post model initiation by tamoxifen administration in locations such as the back. This HO can expand over time and new lesions can form in close proximity, mirroring the expansions of the heterotopic bone field seen in human FOP. (C) In addition to the back, HO develops in other locations seen in FOP such as the limbs, sternum, ribcage, jaw, and hip. The location of each lesion is pinpointed by yellow arrows.

In order to understand how ACVR1[R206H] drives HO in FOP and to ascertain whether this pathological process is ligand-dependent, we developed a conditional-on knock-in mouse model of the R206H variant. The decision to generate a conditional model (wherein the disease-causing allele is hidden and only rendered accessible when certain conditions are met) was based on observations that a previously generated unregulated knock-in mouse line – *Acvr1*^*tm1Emsh/+*^ (www.informatics.jax.org/allele/MGI:5471642) – could not be propagated past the F1 generation due to early postnatal lethality [18]. The engineering and phenotypic characterization of our mouse model – *Acvr1*^*[R206H]FlEx/+*^ (*Acvr1*^*tm2.1Vlcg*^; MGI:5763014) – have been described [1] and hence will be reviewed here only briefly (Fig. 1A). In order to impart conditionality, the R206H variant was encoded in exon 5 of *Acvr1*, and the resulting mutant exon was placed in the antisense orientation within intron 5 of *Acvr1*. Simultaneously, a wild type exon 5 from human *ACVR1* was placed upstream of the mutant exon 5 (which is in the antisense strand) in order to preserve the structure of the resulting transcript. These elements – the wild type exon 5 and mutant exon 5 – were flanked by FlEx arrays [29] in a manner such that upon action of Cre, the wild type exon is deleted and mutant exon 5 is brought into the sense strand. As a result, Cre converts the conditional-on knock-in *Acvr1*^*[R206H]FlEx/+*^ mice to *Acvr1*^*[R206H]/+*^ mice, which genotypically mirror FOP patients. As with most conditional lines, *Acvr1*^*[R206H]FlEx/+*^ mice can be utilized with any Cre driver such as tissue specific Cre drivers (to query the functions of Acvr1[R206H] in specific cell types) and/or small molecule-regulated Cre drivers (to dissect the functions of Acvr1[R206H] at specific time points during development and postnatal life) [30]. As our mouse model has been shared with other labs conducting research in FOP, examples of both types of uses have already been realized [3,31]. In our experiments, we routinely employ the Cre driver *Gt(ROSA26)Sor*^*CreERt2*^, as it ubiquitously expresses a version of Cre – CreERt2 – that remains inactive until activated by tamoxifen [32,33]. Consequently, the perinatal lethality experienced with *Acvr1*^*tm1Emsh/+*^ mice [18] is bypassed, while mice that genotypically recapitulate human FOP can be easily generated.

After treatment with tamoxifen, *Acvr1*^*[R206H]FlEx/+*^; *Gt(ROSA26)Sor*^*CreERt2*^ mice develop progressive heterotopic ossification (Fig. 1B) at anatomic locations mirroring those observed in FOP [1,3] (Fig. 1C), albeit with kinetics that do not necessarily mirror those that have been recorded in human FOP. This data is in agreement with observations made using *Acvr1*^*tm1Emsh/+*^ mice [18]. Heterotopic bone lesions form at the base of the skull and paraspinally (Fig. 1B), as well as on the sternum, rib cage, jaw, and limbs (Fig. 1C), all locations that have been documented in human FOP. During the course of our experiments we detected the emergence of unprovoked HO (what has been traditionally referred to as “spontaneous” HO), however we also noted that HO often developed more consistently in regions subjected to increased handling. Therefore, we routinely employ some type of insult (e.g. handling, muscle injury, local injection of either cardiotoxin, adenovirus, or tamoxifen into the calf muscle, surgery) in order to generate cohorts in which the emergence of HO is uniform across individual mice from the standpoint of both timing and location. This is particularly important when evaluating the efficacy of pharmacological interventions [1,3,31], as well as in other studies such as gene expression profiling and biomarker discovery, primarily because it reduces variability and, by extension, the number of mice needed per study.

### 2.2. The process by which Acvr1[R206H] causes FOP is dependent on ligand

With access to a mouse model that appears to be a true counterpart of human FOP, we went on to decipher whether Acvr1[R206H] requires activation by ligand(s) in order to drive HO in FOP. As we wanted to take as much of an unbiased approach as possible, in our initial experiments we made use of two very broad inhibitors of BMP ligands, ACVR2A-Fc and ACVR2B-Fc, which are comprised of the extracellular domain of ACVR2A and ACVR2B, fused to the constant region – Fc – of human IgG1 [34–36]. These two broadly-acting BMP inhibitors were indeed capable of blocking HO when used prophylactically either alone or in combination [1]. This indicated that it was very likely that somewhere along the process of HO in murine FOP, Acvr1[R206H] requires ligand(s); hence, we proceeded to identify them.

**Fig. 2.**
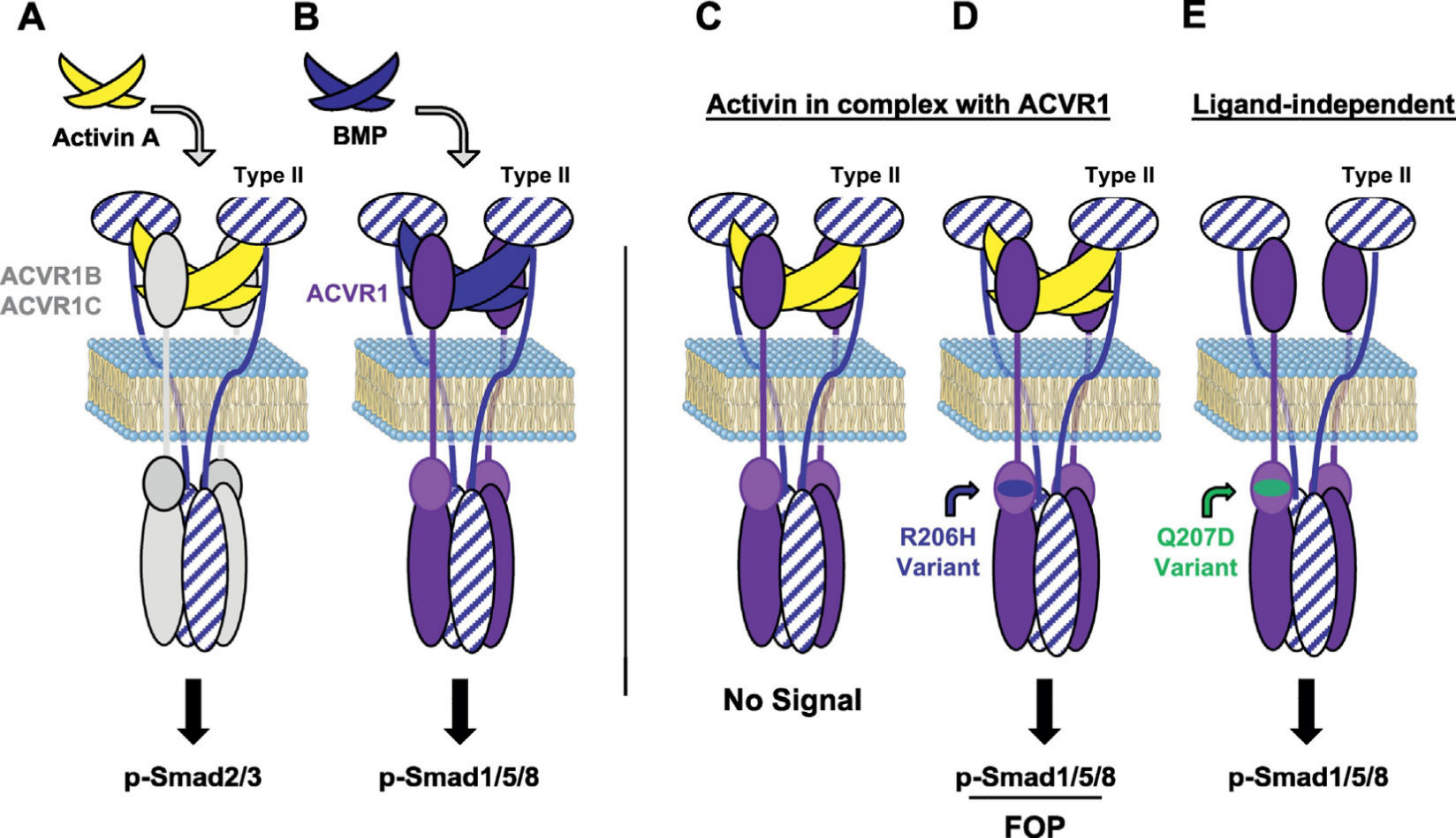
ACVR1[R206H] has gained the ability to recognize Activin A as an agonist. (A) Activin A signals via the type I receptors ACVR1B/1C, inducing phosphorylation of Smad2/3, yet shares type II receptors (ACVR2A, ACVR2B, and BMPR2) with BMPs. (B) BMPs do not utilize ACVR1B/1C as type I receptors; they signal through ACVR1 in complex with ACVR2A, ACVR2B, and BMPR2 to induce phosphorylation of Smad1/5/8. (Note that BMPs also form complexes with other type I receptors - in our Review we focusing mainly on ACVR1.) (C) ACVR1, in conjunction with the type II receptors, binds Activin but the resulting complex does not stimulate phosphorylation of Smad1/5/8; instead, Activin acts as a competitive inhibitor of canonical BMP-mediated signaling through ACVR1. (D) In FOP, when ACVR1[R206H] is engaged by Activin (in the context of the type II receptors), the resulting receptor complexes induce Smad1/5/8 phosphorylation. Hence, ACVR1[R206H] recognizes Activin A just like a BMP, effectively converting the typeII•ACVR1•Activin complex from a ‘dead end’ complex into a signaling complex. These results have been extended to all of the FOP-causing ACVR1 variants described to date [2] (unpublished results). Neither ACVR1[R206H] nor any of the other FOP-causing ACVR1 variants lose their ability to respond to canonical BMPs. (Note: The type II•ACVR1•Activin complex shown here comprises a heterodimer of ACVR1•ACVR1[R206H]; however, this is not an obligate arrangement – homodimers of ACVR1[R206H] also transduce signal.) (E) An artificially generated variant commonly used in experiments – ACVR1[Q207D] – is constitutively active, and hence turns on Smad1/5/8 phosphorylation in the absence of engagement by ligands [51]. Activin A is not able to inhibit ACVR1[Q207D] from signaling nor can it stimulate it further (unpublished results).

As it would have been impractical (if not nearly impossible due to lack of reagents) to test inhibition of each BMP family member recognized by ACVR2A-Fc and ACVR2B-Fc, we first asked whether any of the ligands inhibited by ACVR2A-Fc and ACVR2B-Fc [35,36] are regulated by inflammation. Most prominent were the Activins as they have been shown to be expressed by innate immune system cells during inflammation and to play a role in both promoting and resolving inflammation [37, 38]. This association was appealing, as it provided a potential connection between inflammation and HO; however, it also posed two conundrums: First, unlike osteogenic BMPs, none of the Activins had ever been shown to possess either chondrogenic or osteogenic properties. Second, it had been demonstrated that although Activins bind ACVR1 [39–41] they cannot activate this receptor and do not induce phosphorylation of Smad1/5/8 [42]; instead, Activins utilize the type I receptors ACVR1B (ALK4), and ACVR1C (ALK7) to initiate signaling primarily via Smad2/3 (Fig. 2A) [42,43].)

### 2.3. ACVR1[R206H] has gained the ability to respond to Activin A

**Fig. 3.**
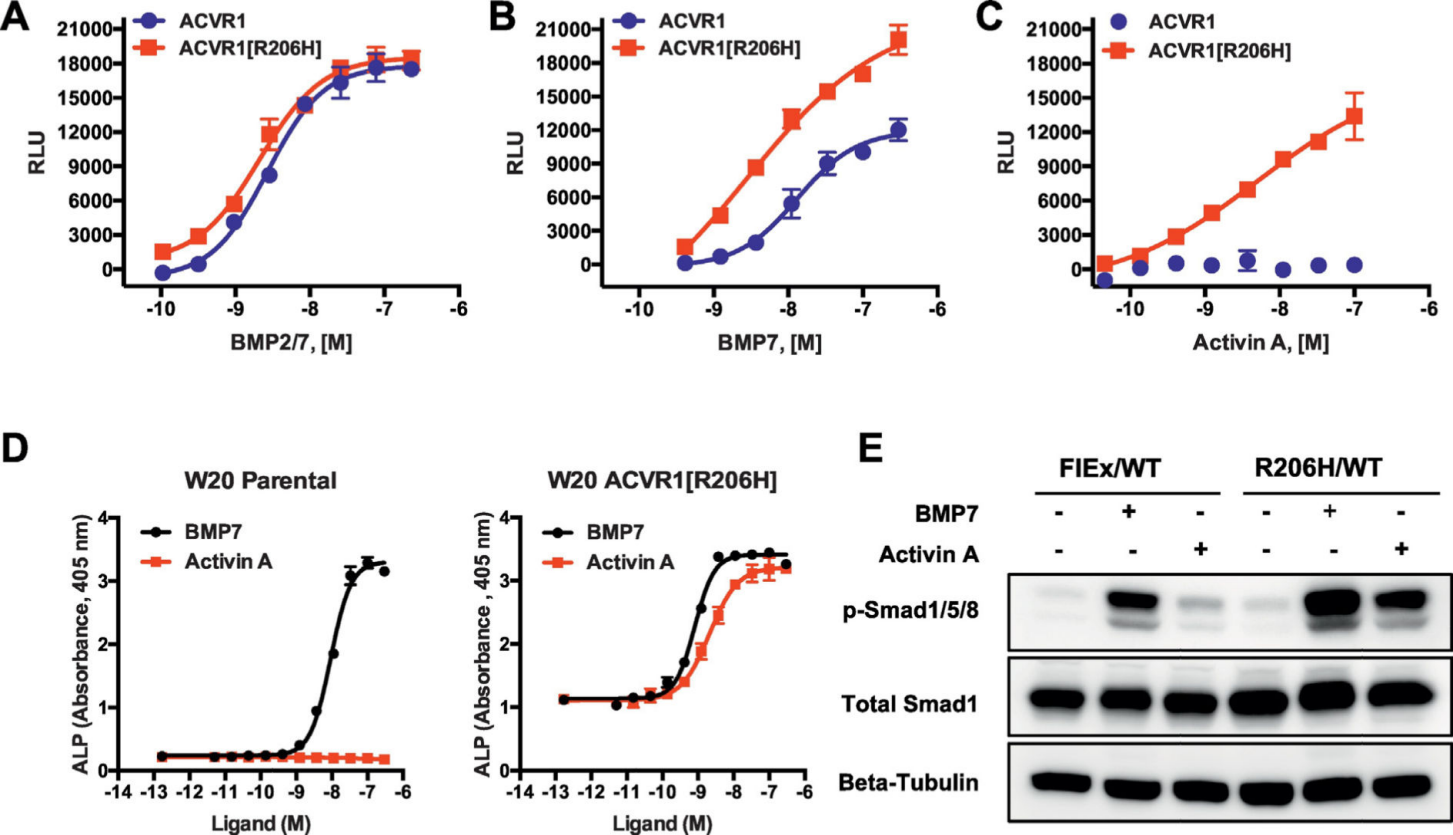
Activin A activates ACVR1[R206H] signals through Smad1/5/8 in human and mouse cells, and hence acts much like a BMP. (A, B, C) The responsiveness of ACVR1 or ACVR1[R206H] to different BMP family ligands was tested in HEK293 cells overexpressing either ACVR1 or ACVR1[R206H] and the Smad1/5/8 reporter BRE-luciferase [1]. (A) ACVR1[R206H] and ACVR1 respond equally to some BMPs, including BMP2/7. (B) ACVR1[R206H] displays an enhanced response to some ligands, e.g. BMP7. (C) ACVR1[R206H] responds to Activin A just like it does to a BMP, whereas ACVR1 does not recognize Activin A as an agonistic ligand. (D) Identical results were obtained in the mouse bone marrow stromal cell line W20, expressing either endogenous ACVR1 or overexpressing ACVR1[R206H] and using alkaline phosphatase (ALP) as a readout for activation of Smad1/5/8 signaling. (E) The same pattern is observed in a non-overexpressing system, i.e. mouse embryonic stem (ES) cells, in which the R206H variant has been knocked in (as described in Fig. 1A). Mouse ES cell line *Acvr1*^*[R206H]FlEx/+*^; *Gt(ROSA26)Sor*^*CreERT2/+*^ (FlEx/WT) and its post-Cre counterpart, ES line *Acvr1*^*[R206H]/+*^; *Gt(ROSA26)Sor*^*CreERT2/+*^ (R206H/WT) were treated with 6 nM BMP7 or 6 nM Activin A for 1 h prior to protein lysate collection. pSmad1/5/8 was measured by Western blotting using beta-tubulin as a loading control. Mirroring results obtained in HEK293 cells and W20 cells, R206H/WT ES cells recognize Activin A as an agonistic ligand, whereas FlEx/WT cells do not. In contrast, both lines respond to BMP7. A more detailed presentation of the responsiveness of ACVR1[R206H] to BMP family ligands can be found in Hatsell, Idone et al. [1] and Dey et al. [3]. The responsiveness of other FOP-causing variants of ACVR1 to Activin A and other BMP family ligands has been examined in detail by Hino et al. [2]. The data presented here was generated as described [1].

Nonetheless, we tested whether Activin A, B, AB, and AC could activate signaling from ACVR1[R206H] and compared it with wild type ACVR1 in several different mouse and human cell lines, both by overexpression (in HEK293 and W20 cell lines), as well as using ‘knock-ins’ of the R206H variant in mouse and human ES cells. In agreement with previous reports, none of these Activins activated wild type ACVR1 in any of the lines tested. In contrast, ACVR1[R206H] recognized all of these Activins as agonistic ligands, and responded to them just as it would to a BMP [1] (Figs. 2D, and 3). These results were independently verified soon thereafter and also extended to the majority of FOP-causing variants of ACVR1 [2] (unpublished results). Recently, this “neofunction” of ACVR1[R206H] to perceive Activin A just like a BMP has been extended to the tissue-resident progenitors that give rise to HO in FOP [3], as is discussed in more detail below. Still, these findings remain surprising as the FOP-causing variants of ACVR1 all arise from mutations in the region of ACVR1 that encodes for its intracellular domain, and hence are unlikely to alter the binding of ligands (Fig. 2). Moreover, these findings indicated that Activins are indeed physiological ligands for ACVR1, but that instead of acting as agonists (turning on signaling), they act as antagonists: they generate ‘dead-end’ complexes between ACVR1 and the type II receptors ACVR2A or ACVR2B (and perhaps also BMPR2), consequently tying up ACVR1 along with its partner type II receptors into inactive complexes (in the presence of Activin) and preventing the utilization of these receptors by other, signal-inducing, BMPs (Fig. 2C). This indeed appears to be the case in vitro [1,44]; however, the physiological relevance of these findings remains unexplored.

### 2.4. Activin A is an obligate ligand for driving HO in FOP

In order to determine whether the ability of Activin A to activate FOPcausing variants of ACVR1 bears any relevance to the pathophysiology of FOP, and particularly HO, we tested whether Activin A can induce the formation of heterotopic bone in *Acvr1*^*[R206H]/+*^ mice, using a classic osteogenic assay wherein the ligand is adsorbed into collagen sponges, implanted intramuscularly, and the formation of bone within and around the implants is monitored [16]. Not surprisingly, Activin A could not induce bone formation in the implants placed in wild type mice. In contrast, Activin A was as efficient as BMP2 (a bona fide osteogenic BMP) at inducing bone formation in the implants introduced in *Acvr1*^*[R206H]/+*^ mice [1]. This demonstrated that Activin A can signal just like an osteogenic BMP via Acvr1[R206H], but it did not prove that Activin A is the physiological ligand for initiating HO in FOP. To prove this, we tested whether inhibition of Activin A could prevent HO. Prophylactic treatment of our mouse model of FOP with a highly specific monoclonal antibody (mAB) to Activin A (as well as Activin AB, and AC) resulted in complete inhibition of HO in the overwhelming majority of mice treated. Inhibition of Activin A using an anti-Activin A mAB has been effective irrespective of whether heterotopic ossification was ‘spontaneous’ (i.e. could not be ascribed to any particular insult), or experimentally-induced by repeated localized injury to muscle or tendons (Hatsell et al., unpublished data). Taken together, these results demonstrate that Activin A is an obligatory secreted factor at least during the initiation of HO in FOP, and further indicate that ACVR1[R206H] has to be activated by a ligand – Activin A – in order to cause HO. These results do not inform us about the role of Activin A once heterotopic bone has started to form, but we anticipate that Activin A ceases to play a role once the heterotopic bone has been established and connected to the skeleton, as by that time it is effectively indistinguishable from normal bone [45,46]. These results also do not inform whether Activin A plays a role during the pre-osseous stages of a developing lesion; this is an area that we are currently exploring. Nonetheless, given the effectiveness of Activin A inhibition in stopping the formation of HO, we are undertaking the development of an anti-Activin A neutralizing antibody as a potential therapy for FOP (NCT02870400 and NCT03188666; see also Discussion section).

### 2.5. Identification of tissue-resident progenitor cells that give rise to HO

The discovery that Activin A is an obligatory player for the formation of heterotopic bone in FOP still left unidentified the cells that generate heterotopic bone. Identification of those cells would enable examination of the aberrant signaling of ACVR1[R206H] *in physiologically relevant cell types* – i.e. in the cells that nucleate the formation of the heterotopic bone – and hence perhaps uncover additional therapeutic targets. Not surprisingly, identification of the cells that form heterotopic bone in FOP had already been an active area of investigation, with several publications proposing mesenchymal cells [47], Tie2-expressing “endothelial precursors” [48], vascular endothelial cells [26], and lastly a *CD31*^*−*^*CD45*^*−*^ *PDGFRa*^*+*^*Sca1*^*+*^ interstitial muscle-resident progenitor [49] as the cells that drive HO. However, all of these studies were performed in mouse models that were not genotypically accurate reflections of FOP; instead they utilized transgenic lines overexpressing BMP4 (Nse-BMP4 transgenic line, Tg(Eno2-Bmp4)3Jake) [47,48], implants of endothelial cells expressing ACVR1[R206H] [26], or BMP2-induced HO generated by intramuscular implantation of BMP2-impregnated matrigel [48,49].

The availability of an accurate mouse model of FOP facilitated the search for the progenitor cells that give rise to heterotopic bone specifically in FOP. Such progenitors should have several properties: (**a**) They should reside in the tissues where heterotopic bone forms in FOP (i.e. skeletal muscle, tendons, ligaments); (**b**) they should express ACVR1 and, as long they are *ACVR1*^*R206H/+*^, be able to respond to Activin A and turn on Smad1/5/8 signaling; and (**c**) when they respond to Activin A they should assume a chondrogenic fate. In a set of experiments published in 2016, Dey and co-investigators initially utilized a transgenic line conditionally overexpressing ACVR1[Q207D] – Tg(CAG-LacZ,- ACVR1*,-EGFP)35–1Mis [50] – in conjunction with a set of tissue-specific Cre transgenes, to query the possible sources of progenitors cells for their ability to generate HO. ACVR1[Q207D] is an engineered variant that is constitutively active (and effectively signals independent of ligands) [51,52] (Fig. 2E). ACVR1[Q207D] has not been associated with FOP; however, because the aforementioned conditional transgenic line had been shown to be very efficient in generating HO [53], it was considered as a sensitive way to screen for this phenotype. It also turned out to be a serendipitous choice as it helped uncover a role for injury (see below).

This screen tested which of eight different cell lineages that had been previously implicated as sources of HO actually go on to form HO as a result of activation of Smad1/5/8 signaling. Of these eight lineages, only two could form heterotopic bone: *Mx1*^*+*^*Lin*^*−*^*Sca1*^*+*^*PDGFRa*^*+*^ progenitors that reside in muscle interstitium and drive injury-dependent HO in skeletal muscles, and *Scx*^*+*^*Lin*^*−*^*Sca1*^*+*^*PDGFRa*^*+*^ progenitors that reside in ligaments and tendons and drive unprovoked (“spontaneous”) HO in those tissues [3]. The ability of these two progenitor cells to give rise to HO was verified in the genetically and physiologically more accurate model, *Acvr1*^*[R206H]FlEx/+*^.

In vivo, these *Mx1*^*+*^ and *Scx*^*+*^ progenitors give rise to nearly all of the chondrocytes that form the cartilage anlagen that go on to become heterotopic bone lesions, but do so only if they are *Acvr1*^*R206H/+*^, or if they transgenically express ACVR1[Q207D]. It is important to stress that the location of these two progenitors is tissue-restricted; in contrast, *Acvr1* is widely expressed by many different cell types in many other tissues (http://www.informatics.jax.org/marker/MGI:87911), the majority of which are not sites where HO arises in FOP. Hence, the ‘tissue-residency’ of these two different progenitors appears to define the anatomical sites where HO can occur in FOP.

Ex vivo, these progenitors differentiate towards a chondrocytic lineage in response to Activin A and in a Smad1/5/8-dependent manner but only if they are *Acvr1*^*R206H/+*^. In contrast, wild type progenitors do not adopt a chondrocytic fate when treated with Activin A. These results demonstrated for the first time that the aberrant response to Activin A operates in the progenitors that differentiate into the chondrocytes that nucleate HO in FOP. Together with the finding that Activin A is the main physiologic driver of HO in FOP (via mutant ACVR1), these results effectively confirmed our initial hypothesis that the process of HO in FOP is ligand-dependent. Furthermore, also in line with our original hypothesis, HO in FOP involves a ligand that not only activates ACVR1[R206H] (and all the other FOP-causing variants of ACVR1) but it is also regulated by inflammation, and recognized by tissue-resident progenitor cells that can give rise to heterotopic bone via an endochondral process.

## 3. Discussion

The discovery of ACVR1 – a type I BMP receptor – as the causative gene in FOP acted as a catalyst of studies aimed at understanding the molecular and cellular aspects of the pathophysiology of this disease and also pinpointed ACVR1 as a new therapeutic target (see below). Although initial work indicated that ACVR1[R206H], as well as other FOP-associated variants of ACVR1, cause HO because they exhibit enhanced signaling either in the apparent absence or in the presence of certain BMPs (reviewed in [54]), it turned out that the key mechanism had been missed: FOP-causing ACVR1 variants drive HO in FOP by perceiving their own natural antagonist – Activin A – as an agonist, i.e. just like an osteogenic BMP. Therefore, the ability of Activin A to bind ACVR1 and effectively antagonize signaling from that receptor, is hijacked in FOP and used for the opposite purpose: to activate signaling through the FOP-causing ACVR1 variants. It is currently unclear whether BMPs also play a role in the formation of heterotopic bone in FOP – this possibility has not been formally excluded, either by genetic ablation or pharmacologic inhibition of candidate BMPs [55] in mouse models of FOP. It is equally unclear whether Activin A is involved in the patterning defects that have been described in some FOP patients [4,6–8]. However, given that one of the most debilitating aspects of FOP is the cumulative heterotopic bone and its associated comorbidities, the fact that Activin A directly acts on mutant ACVR1 to trigger HO in FOP indicates that this is an obligatory pathophysiologic mechanism, and moreover one with implications for the development of therapeutics.

### 3.1. The role of tissue damage in HO

As surprising as the discovery of this role for Activin A was, it turned out that it was only part of the story; experiments with a ligand-independent constitutively-active (and effectively ‘maxed-out’ from the standpoint of inducing BMP signaling) artificial variant – ACVR1[Q207D] – showed that activation of BMP signaling alone was not capable of inducing the formation of heterotopic bone in skeletal muscles – injury or some other type of tissue damage was also required [3]. The mechanisms by which tissue damage participates in HO in FOP (beyond the recruitment of an immune cell infiltrate [1,14–16]) are unclear. Nonetheless, the fact remains that the Mx1^+^ progenitors cannot proceed down the chondrocytic lineage until they receive a Smad1/5/8 activation signal. In the case of FOP, that signal is Activin A (as its inhibition completely prevents the occurrence of HO [1]). In contrast, in the case of non-genetic or non-syndromic forms of HO – wherein HO is not driven by mutations in ACVR1 – the responsible signals appear to be the classically osteogenic BMPs [49,56]. Given this apparent requirement of osteogenic BMPs for non-genetic HO, the lack of an obvious contribution of BMPs in HO in FOP (at least during the initial stages of HO) it is our opinion that any BMPs present play a secondary role in that process. Alternatively, it is possible that in the region of an emerging lesion in FOP, BMPs are not expressed at high levels, whereas in severe trauma-induced HO they are. This idea is supported by the fact that in a non-severe trauma setting, exogenous BMP2 is required to drive the differentiation of the *Mx1*^*+*^ progenitors down the path of endochondral ossification [49], whereas there is no such requirement under conditions of severe trauma [56]. Reciprocally, Activin A does not appear to play an important role in non-genetic HO – we have recently completed a set of in vivo experiments that show that inhibition of Activin A has no effect on the development of traumainduced HO (unpublished results). Hence, it appears that it is only in FOP that Activin A is interpreted as a HO-inducing factor because of the ability of ACVR1[R206H] (and other FOP-causing variants of *ACVR1*) to perceive Activin A just like an osteogenic BMP.

### 3.2. A working model for HO in FOP

**Fig. 4.**
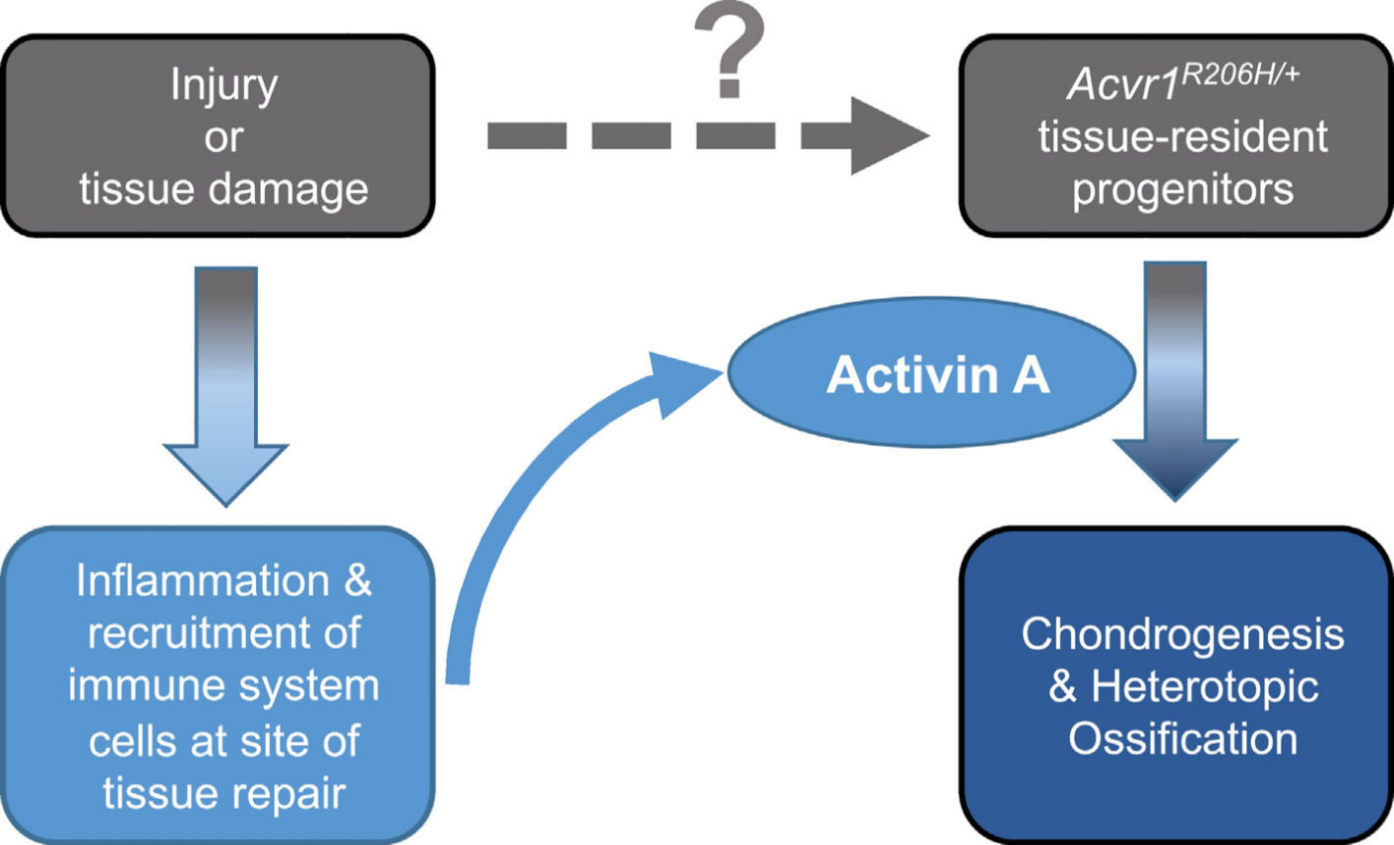
A working model for the formation of heterotopic bone in FOP. Tissue damage to skeletal muscles, ligaments, and tendons as a result of injury or regular use results in the recruitment of immune system cells into the area in need of repair. In addition, tissue damage provides a yet-to-be-identified signal that enables tissue-resident progenitor cells to become competent to give rise to the chondrocytes that form the initial cartilage anlagen that will give rise to the heterotopic bone. Activin A, secreted by the immune system cells at the site of tissue damage, is perceived as a pro-osteogenic signal by the tissue-resident progenitor cells. (Alternatively, the immune system cells may instruct other cells in the area of the future heterotopic bone lesion to express Activin A.) These progenitor cells, in response to Activin A, differentiate into chondrocytes and proceed to form heterotopic bone lesions.

Taken together, these findings lead us to a model that describes how HO emerges in FOP. This model provides a synthesis of five key findings in FOP research: (**a**) The discovery of the causative gene [57]; (**b**) the presence of immune cell infiltrates in the early lesion [1,14–16,18]; (**c**) the requirement for the kinase activity of ACVR1 for the formation of HO [3]; (**d**) the obligatory roles of both Activin A [1] and injury or tissue damage [3]; and, (**e**) the identification of the progenitor cells that respond to Activin A and initiate the formation of heterotopic bone yet only if they are *Acvr1*^*[R206H]/+*^ [3]. In this model (Fig. 4) localized injury or tissue damage (i.e. tissue repair-inducing stimuli) cause infiltration of the repairing site by immune system cells, and – at least in muscle – may contribute in other ways to enable HO. The immune system cells that migrate to the site of tissue damage introduce Activin A (or perhaps cause the expression of Activin A by other cell types in the area of the future lesion), which in turn causes the progenitors to differentiate into chondrocytes, hence nucleating the cartilage anlagen that progress to heterotopic bone.

### 3.3. Open questions, future directions

This model accounts for the major findings in FOP to date, but it also serves to highlight the gaps in our current understanding. It is not our objective to provide an exhaustive list of the questions still surrounding the pathophysiology of FOP; rather we aim to highlight those that directly pertain to our findings. Most obvious is perhaps the fact that we still do not know whether BMPs play *any* role in the formation of heterotopic bone in FOP. Our initial experiments with ACVR2B[L79D]-Fc – a variant of ACVR2B-Fc that does not block Activin A [35] – showed that ACVR2B[L79D]-Fc had no effect on HO in FOP mice (unpublished results), indicating that the ligands inhibited by it do not play a significant role in FOP. ACVR2B[L79D]-Fc however does not inhibit osteogenic BMPs such as BMP2 and BMP4; hence, the role of these BMPs in HO in FOP remains to be tested.

Perhaps more important for understanding the events that lead to the initiation of HO is the exact origin of Activin A in the developing lesion. Activin A is expressed by innate immune system cells during the initial stages of inflammation and tissue repair [37,38]. However, there is only preliminary evidence of Activin A expression by these type of cells during the stages of a developing heterotopic bone lesion that precede the formation of chondrocytes (unpublished results).

From a molecular mechanism standpoint, one of the main questions that remains is how intracellular mutations in ACVR1 alter the outcome of its engagement by Activin: wild-type ACVR1 utilizes Activin as a competitive inhibitor of BMP-induced signaling (by tying up ACVR1 in a non-signaling complex with the type II receptors [1,44]); whereas, FOP-causing variants of ACVR1 fail to distinguish between Activins and BMPs and perceive both classes of ligands as agonists [1,2]. Although reduced binding of FKBP12 to mutant ACVR1 has been proposed as a potential mechanism [25,58–59] our results to date show that this is not the case [1] (unpublished results), in agreement with emerging data from structural studies [60].

Lastly, connecting back to in-patient observations, one of the gaps in our knowledge concerns the variability in the expressivity of FOP even between patients that share the same variant – *ACVR1[R206H]*. Unlike our mouse model (which is largely inbred and displays a rather predictable response to injury by forming HO within 2 to 3 weeks), the severity and progression of HO in human FOP appears to be embellished by genetic modifiers. Of particular interest would be those that drive rapid progression of HO, versus those that inculcate milder or slow-progressing disease. Discovery of these modifiers can be attempted in animal models and may lead to a better understanding of FOP and even provide prognostic biomarkers that can be used in making decisions about therapy. Along the same lines, it is unclear why in FOP patients there is a subset of instances in which HO does not form as a result of injury [6,10,61]. This is a phenomenon that we have not been able to ascertain in mice but it suggests that there may be ways by which the process that leads to HO can be resolved prior to the generation of mineralized lesions.

### 3.4. ACVR1, Activin A, and drug development in FOP

In spite of these open questions, drug discovery research in FOP has made great progress in the last few years. There has been long-standing interest in developing drugs for this genetic disorder, even preceding the discovery of ACVR1 as the causative gene. Early efforts focused on the endochondral ossification process, as it can be blocked by noggin [16] or by Retinoic Acid Receptor Gamma (RARG) agonists [62–64]. In fact, one such agent – Palovarotene [65] – has been repositioned into the FOP space and is undergoing clinical testing (NCT02979769, NCT02279095, and NCT02190747); Palovarotene was also recently validated in FOP mice [31]. Other targets and possible therapeutic agents have already been discussed in several reviews [54,57] and some have been tested in preclinical models of HO [53,66,67], and occasionally in FOP mice [3].

The identification of *ACVR1* provided an additional and very tractable opportunity for further drug development. This is in part because unlike the case for most genetic disorders, ACVR1 is both a receptor and a kinase, and therefore, provides at least four different ‘ACVR1-centric’ paths to drug development: (**a**) inhibitors of ACVR1 kinase (several of which are under development) [68–72]; (**b**) antisense oligonucleotides or siRNA based therapeutics [73,74]; (**c**) antibodies that bind the extracellular domain of ACVR1 and block its function; and, (**d**) antibodies to putative ligand(s) responsible for activating ACVR1 in a manner that results in HO. Our findings of the obligatory requirement for Activin A for HO in FOP provides an example of the last category. Based on our data, we are in process of developing a neutralizing antibody to Activin A as a potential therapy for FOP (NCT02870400, NCT03188666), while we continue to explore the open questions that we have identified in a continued quest to attain a more complete understanding of the molecular and cellular aspects of the pathophysiology of FOP.
